# Anti-Transforming Growth Factor β IgG Elicits a Dual Effect on Calcium Oxalate Crystallization and Progressive Nephrocalcinosis-Related Chronic Kidney Disease

**DOI:** 10.3389/fimmu.2018.00619

**Published:** 2018-03-29

**Authors:** Stefanie Steiger, Julia Felicitas Grill, Qiuyue Ma, Tobias Bäuerle, Jutta Jordan, Michaela Smolle, Claudia Böhland, Maciej Lech, Hans-Joachim Anders

**Affiliations:** ^1^Division of Nephrology, Medizinische Klinik und Poliklinik IV, Klinikum der Universität München, Munich, Germany; ^2^Preclinical Imaging Platform Erlangen, Institute of Radiology, Friedrich-Alexander-Universität Erlangen-Nürnberg, Erlangen, Germany; ^3^Ludwig-Maximilians Universität München, Biomedizinisches Centrum, Munich, Germany; ^4^Department of Radiation Oncology, Ludwig-Maximilians Universität München, Munich, Germany

**Keywords:** calcium oxalate, crystallization, transforming growth factor β, fibrosis, nephrocalcinosis, chronic kidney disease

## Abstract

Crystallopathies are a heterogeneous group of diseases caused by intrinsic or environmental microparticles or crystals, promoting tissue inflammation and scarring. Certain proteins interfere with crystal formation and growth, e.g., with intrarenal calcium oxalate (CaOx) crystal formation, a common cause of kidney stone disease or nephrocalcinosis-related chronic kidney disease (CKD). We hypothesized that immunoglobulins can modulate CaOx microcrystal formation and crystal growth and that therefore, biological IgG-based drugs designed to specifically target disease modifying proteins would elicit a dual effect on the outcome of CaOx-related crystallopathies. Indeed, both the anti-transforming growth factor (TGF)β IgG and control IgG1 antibody impaired CaOx crystallization *in vitro*, and decreased intrarenal CaOx crystal deposition and subsequent CKD in mice on an oxalate-rich diet compared to oxalate-fed control mice. However, the TGFβ-specific IgG antibody showed nephroprotective effects beyond those of control IgG1 and substantially reduced interstitial fibrosis as indicated by magnetic resonance imaging, silver and α-smooth muscle actin staining, RT-qPCR, and flow cytometry for pro-fibrotic macrophages. Suppressing interstitial fibrosis slowed the decline of glomerular filtration rate (GFR) compared to treatment with control IgG1 [slope of *m* = −8.9 vs. *m* = −14.5 μl/min/100 g body weight (BW)/day, Δ = 38.3%], an increased GFR at the end of the study (120.4 vs. 42.6 μl/min/100 g BW, Δ = 64.6%), and prolonged end stage renal disease (ESRD)-free renal survival by 10 days (Δ = 38.5%). Delayed onset of anti-TGFβ IgG from day 7 was no longer effective. Our results suggest that biological drugs can elicit dual therapeutic effects on intrinsic crystallopathies, such as anti-TGFβ IgG antibody treatment inhibits CaOx crystallization as well as interstitial fibrosis in nephrocalcinosis-related CKD.

## Introduction

Crystal deposition is relatively common in the kidneys and is often associated with inflammation, tubular injury, and interstitial fibrosis (1). Crystals of calcium oxalate (CaOx) are commonly found in kidney stone disease (1) accounting for approximately 80% of all types of kidney stones and can cause chronic kidney disease (CKD) (2). Unlike symptomatic urolithiasis, intrarenal nephrocalcinosis is often asymptomatic, but can lead to significant kidney injury and renal failure (3–5). The mechanism of CaOx crystal formation involves a combination of processes including urine supersaturation of stone-forming salts, such as calcium and oxalate, urinary pH, and lack of crystallization inhibitors in the urine (6, 7). These crystallization inhibitors like nephrocalcin, osteopontin (8), Tamm–Horsfall glycoprotein (9), and uropontin (10) have been identified in the urine of healthy individuals but they are decreased in kidney stone formers. Interactions between CaOx crystals and the tubular compartment evoking an inflammatory response associated with the release of pro-inflammatory mediators, cell death, and leukocyte infiltration, which further contributes to tubular atrophy and interstitial fibrosis, leading to progressive nephrocalcinosis (11). We first speculated that proteins potentially suitable for therapy in humans could have a similar protective effect on CaOx crystallization, e.g., immunoglobulin (IgG).

Therapies to prevent renal failure from CaOx crystal-induced nephropathy and/or nephrocalcinosis have been directed principally at lowering serum and urine oxalate, the use of an oxalate-reduced diet and calcium supplementation in patients with enteric hyperoxaluria, as well as anti-inflammatory therapies (12). CKD progression in nephrocalcinosis is associated with profound interstitial fibrosis (13), a histopathological feature of kidney atrophy thought to contribute to CKD progression (14). Transforming growth factor (TGF)β is a critical mediator of organ fibrosis and has been repeatedly validated as a molecular target in disease (15). However, in renal fibrosis only few studies assessed glomerular filtration rate (GFR), a clinically relevant marker of renal excretory function, as an endpoint in studies with interventions modeling renal fibrogenesis.

We hypothesized that anti-TGFβ IgG may not only inhibit interstitial fibrosis but also influence the crystallization of CaOx inside the kidney, which both should synergize to prevent nephrocalcinosis-related GFR decline, i.e., CKD progression. To address this concept, we employed a mouse model of progressive CaOx crystal-driven CKD (13) with preemptive or delayed anti-TGFβ IgG treatment (16, 17).

## Materials and Methods

### Animal Studies

Eight-week old male C57BL/6N mice were obtained from Charles River Laboratories (Sulzfeld, Germany). Mice were housed in groups of five in filter-top cages and had access to food and water *ad libitum*. Cages, nest lets, food, and water were sterilized by autoclaving before use. Oxalate-rich diet was prepared by adding 50 µmol/g sodium oxalate to a calcium-free standard diet (Ssniff, Soest, Germany) as previously described (13, 18). Mice were split into four groups (*n* = 5 mice per group): the first group received a control diet without sodium oxalate (control), the second group was injected intraperitoneally (i.p.) with the murine control IgG1 monoclonal antibody (isotype-matched control 13C4 antibody), the third group with the murine IgG1 monoclonal anti-TGFβ antibody that neutralizes all three TGFβ isoforms (1D11, both antibodies kindly provided by Genzyme Corporation, Sanofi, Framingham, MA, USA) 1 day after starting the oxalate-rich diet every alternate day [1.5 mg/kg body weight (BW), total of seven injections] (16, 19), and the fourth group received an oxalate-rich diet only (oxalate only). Serum and urine samples were collected as well as GFR measured from all experimental and control groups on day 0 and before sacrifice by cervical dislocation on day 7 or 14. Urine samples were acidified immediately after collection for oxalic acid estimations. Kidneys were harvested after sacrifice. One kidney was used for flow cytometry analysis and the other was divided into two equal parts. One part was kept in RNA later solution at −80°C for RNA isolation and the second part was kept in 4% formalin to be embedded in paraffin for histology analysis.

For delayed IgG1 or anti-TGFβ antibody treatment, all mice were put on a control diet (*n* = 5) or an oxalate-rich diet (*n* = 5) and two groups of mice received additionally either the control IgG1 antibody (i.p., 1.5 mg/kg BW, *n* = 5) or the anti-TGFβ antibody (i.p., 1.5 mg/kg BW, *n* = 5) four times starting from day 7 every alternate day until sacrifice on day 14 (Figure S1 in Supplementary Material).

### Assessment of Kidney Injury

Kidney sections of 2 µm were stained with Pizzolato to visualize CaOx crystal deposition, which was quantified (% area) using ImageJ software as described previously (20). Periodic acid-Schiff (PAS) reagent was used to assess kidney injury, which was scored by assessing the percentage of atrophic tubules. CD3+ T cells and F4/80+ macrophages (both Serotec, Kidlington, UK) were identified by immunostaining. Fibrotic areas were identified by immunostaining for silver and α-smooth muscle actin (α-SMA) (Dako GmbH, Hamburg, Germany). Quantification of immunostaining (% area) was done using ImageJ software. An observer blinded to the experimental condition performed all assessments. Serum blood urea nitrogen (BUN) (DiaSys, Holzheim, Germany), serum and urine oxalic acid (oxalate) (Libios, Pontcharra-sur-Turdine, France), and urine calcium (Sigma-Aldrich, Taufkirchen, Germany) were measured using commercially available kits as per manufacturer’s protocol.

### Transcutaneous GFR Assessment and Calculation

Glomerular filtration rate measurements were performed in conscious mice on day 0, 7, and 14 (*n* = 5 per group). Briefly, mice were anesthetized with isoflurane to mount a miniaturized imager device built from two light-emitting diodes, a photodiode, and a battery (MediBeacon™ Inc., Mannheim, Germany) onto the shaved neck of the animals (21). The background signal of the skin was recorded for 5 min. Then, mice received a single injection of FITC-sinistrin (i.v., 150 mg/kg BW) (MediBeacon™ Inc., Mannheim, Germany). Each mouse was kept in a single cage and the signal was recorded for 90 min. Data were analyzed using the imaging device MPD Studio software (MediBeacon™ Inc., Mannheim, Germany). GFR (μl/min/100 g BW) was calculated from the decrease of fluorescence intensity of FITC-sinistrin over time using the three-compartment model with linear correction (injection, plasma, and interstitial compartment, *t*1/2 of FITC-sinistrin), BW of mouse, and an empirical conversion factor as per manufacturer’s protocol (21, 22). The slope of daily GFR loss (*m*) was determined with the linear equation using Microsoft Excel

$$\text{GFR }\left[\mu \text{L/min/100 g BW}\right]=\frac{\text{14,616.8 [}\mu \text{L per 100 g BW]}}{t\frac{1}{2}(\text{FITC}-\text{sinistrin})\text{ [minute]}}.$$

### CaOx Crystal Formation *In Vitro*

The formation of CaOx crystals *in vitro* has previously been described in more detail (7). Briefly, 50 µl of a Na_2_C_2_O_4_ solution (oxalate, 0.1 mM, pH 7.3) was mixed with 50 µl CaCl_2_ solution (0.1 mM, pH 7.3) in a 96-well plate at room temperature (RT) for 5 min (CaOx only). To investigate the effect of the IgG1 or anti-TGFβ antibody on CaOx crystal formation, Na_2_C_2_O_4_ solution was pre-incubated with or without the IgG1 or anti-TGFβ antibody (0.2 µg/ml) or an IgG F(ab′)2 fragment antibody (0.2 µg/ml) for 1 h at RT prior to addition of CaCl_2_ buffer (CaOx + IgG1). Different forms of CaOx crystals [CaOx monohydrate (COM) and CaOx dihydrate (COD)] were visualized under a Leica microscopy and quantified using flow cytometry (BD FACSCalibur, Becton Dickinson, NJ, USA).

### RNA Preparation and Real-Time Quantitative PCR

The RNA extraction kit from Qiagen (Düsseldorf, Germany) was used to isolate total RNA from kidneys (*n* = 5 per group) following the manufacturer’s instructions. RNA quality was assessed using agarose gels before being transcribed into cDNA using reverse transcriptase (Superscript II) (Invitrogen, Carlsbad, CA, USA). Real-time RT-PCR was performed using SYBRGreen PCR master mix and analyzed with a Light Cycler 480 (Roche, Mannheim, Germany). All gene expression values were normalized using 18s rRNA as a housekeeping gene. All primers used for amplification were purchased from Metabion (Martinsried, Germany) and are listed in Table 1.

**Table 1:** Murine primer sequences.

Target	Primer sequences
KIM-1	Forward 5′-TCAGCTCGGGAATGCACAA-3′ Reverse 5′-TGGTTGCCTTCCGTGTCTCT-3′
TIMP-2	Forward 5′-CAGACGTAGTGATCAGAGCCAAA-3′ Reverse 5′-ACTCGATGTCTTTGTCAGGTCC-3′
IL-6	Forward 5′-TGATGCACTTGCAGAAAACA-3′ Reverse 5′-ACCAGAGGAAATTTTCAATAGGC-3′
TNFα	Forward 5′-CCACCACGCTCTTCTGTCTAC-3′ Reverse 5′-AGGGTCTGGGCCATAGAACT-3′
ACOX1	Forward 5′-CTTGGATGGTAGTCCGGAGA-3′ Reverse 5′-TGGCTTCGAGTGAGGAAGTT-3′
PGC1α	Forward 5′-AGTCCCATACACAACCGCAG-3′ Reverse 5′-CCCTTGGGGTCATTTGGTGA-3′
PPARα	Forward 5′-TGCAAACTTGGACTTGAACG-3′ Reverse 5′-GATCAGCATCCCGTCTTTGT-3′
TGFβ1	Forward 5′-CAACCCAGGTCCTTCCTAAA-3′ Reverse 5′-GGAGAGCCCTGGATACCAAC-3′
TGFβR1	Forward 5′-GCTCCTCATCGTGTTGGTG-3′ Reverse 5′-CAGTGACTGAGACAAAGCAAAGA-3′
TGFβ2	Forward 5′-CCGCATCTCCTGCTAATGTTG-3′ Reverse 5′-AATAGGCGGCATCCAAAGC-3′
TGFβR2	Forward 5′-GCTGCATATCGTCCTGTGG-3′ Reverse 5′-TCACATCGCAAAACTTGCAC-3′
Collagen1α1	Forward 5′-ACATGTTCAGCTTTGTGGACC-3′ Reverse 5′-TAGGCCATTGTGTATGCAGC-3′
Fibronectin-1	Forward 5′-GGAGTGGCACTGTCAACCTC-3′ Reverse 5′-ACTGGATGGGGTGGGAAT-3′
iNOS	Forward 5′-GAGACAGGGAAGTCTGAAGCAC-3′ Reverse 5′-CCAGCAGTAGTTGCTCCTCTTC-3′
18s RNA	Forward 5′-GCAATTATTCCCCATGAACG-3′ Reverse 5′-AGGGCCTCACTAAACCATCC-3′

### Flow Cytometry Analysis

Kidneys were harvested from mice and then digested in digestion buffer (collagenase/DNase1 solution) for 40 min at 37°C. Digested tissue was passed through a 70 µm filter and washed with cold PBS. For isolating leukocytes, a Nycodenz solution (Axis-Shield, Oslo, Norway) was used to separate CaOx crystals and tissue from renal immune cells. Single cell suspensions were then washed with wash buffer (0.1% BSA, 0.01% sodium azide in PBS) and FcR blocked with anti-mouse CD16/32 (2.4G2) for 5 min. After blocking, cells were stained with the surface antibodies PE/Cy5 anti-mouse CD45 (BioLegend, Fell, Germany), V450 anti-mouse CD11b (BioLegend, Fell, Germany), APC anti-mouse F4/80 (BioRad, München, Germany), FITC anti-mouse CD206 (BD Biosciences, Germany), and PE anti-mouse Cx3CR1 (BD Biosciences, Heidelberg, Germany) for 30 min at 4°C in the dark. Following incubation, cells were washed, centrifuged, and GolgiPlug added for 15 min to avoid release of intracellular cytokines. Cells were then washed, resuspended in cell fixation/permeabilization buffer for an additional 15 min and washed in perm wash buffer. Intracellular antibody for PE/Cy7 anti-mouse TGFβ1 was added to the cell suspension for 40 min at 4°C. After incubation, cells were washed with PBS and reconstituted in 1 ml fresh wash buffer. Flow cytometry analysis was performed using the BD FACSCanto II (Becton Dickinson, NJ, USA) and data analyzed with the software FlowJo 8.7 (Tree Star Inc., Ashland, OR, USA). For determining the absolute number of cells/microlitre, Invitrogen AccuCheck counting beads (Thermo Fisher Scientific, PCB100, Langenselbold, Germany) were used and the absolute cell counts calculated according to manufacturer’s instruction.

### Magnetic Resonance Imaging (MRI)

Kidneys from IgG1- and anti-TGFβ-treated mice with nephrocalcinosis on day 14 (*n* = 4 each group) were harvested and processed in 1.5% agarose gel, and placed in a whole body coil for mice (Bruker BioSpin, Ettlingen, Germany) of a dedicated small animal ultra-high-field magnetic resonance tomograph scanner (ClinScan 7 T, Bruker BioSpin, Ettlingen, Germany). Standard sequences for morphology and mapping of T1 and T2 relaxation times (Siemens, Erlangen, Germany) were performed on kidneys in sagittal orientation. By mapping of relaxation times, specific magnetic properties of tissues are quantified. An increase of T1 relaxation time is associated with fibrosis, while prolonged T2 relaxation times are found in inflammation (13). For post-processing of images, three regions of interest were placed in the cortex, outer and inner medulla, respectively, to determine T1 and T2 relaxation times (Osirix, Bernex, Switzerland, open-source software).

### Microscale Thermophoresis

MST was used to characterize binding affinity between IgG1 and soluble sodium oxalate. Soluble oxalate at various concentrations (1 nM–5 mM) and IgG1 (250 nM) were incubated for 1 h at RT. Measurements were performed with the Monolith NT.LabelFree MST device using standard capillaries (NanoTemper Technologies, Munich, Germany). Buffer only, soluble sodium oxalate or IgG1 only were used as controls. Measurements were performed at 25°C in 67 mM NaPO_4_ buffer, 150 mM NaCl, and 0.05% Tween 20 at pH 7.4. The infrared laser power was between 20 and 40%, and 40–70% LED power was used. A laser on time of 30 s and a laser off time of 5 s were used. Data from the binding assays were analyzed using Nanotemper analysis.

### Statistical Analysis

Statistical analysis was carried out using Student’s *t*-test, two-way ANOVA with Bonferroni’s correction, or one-way ANOVA with Tukey’s comparison and performed using GraphPad Prism5.0 Software. Data are presented as mean ± SEM. Significance was considered to be attained at a value of *p* < 0.05, ns indicates not significant.

## Results

### IgG1 Influences CaOx Crystal Formation Without Binding to Soluble Oxalate *In Vitro*

**Figure 1:**
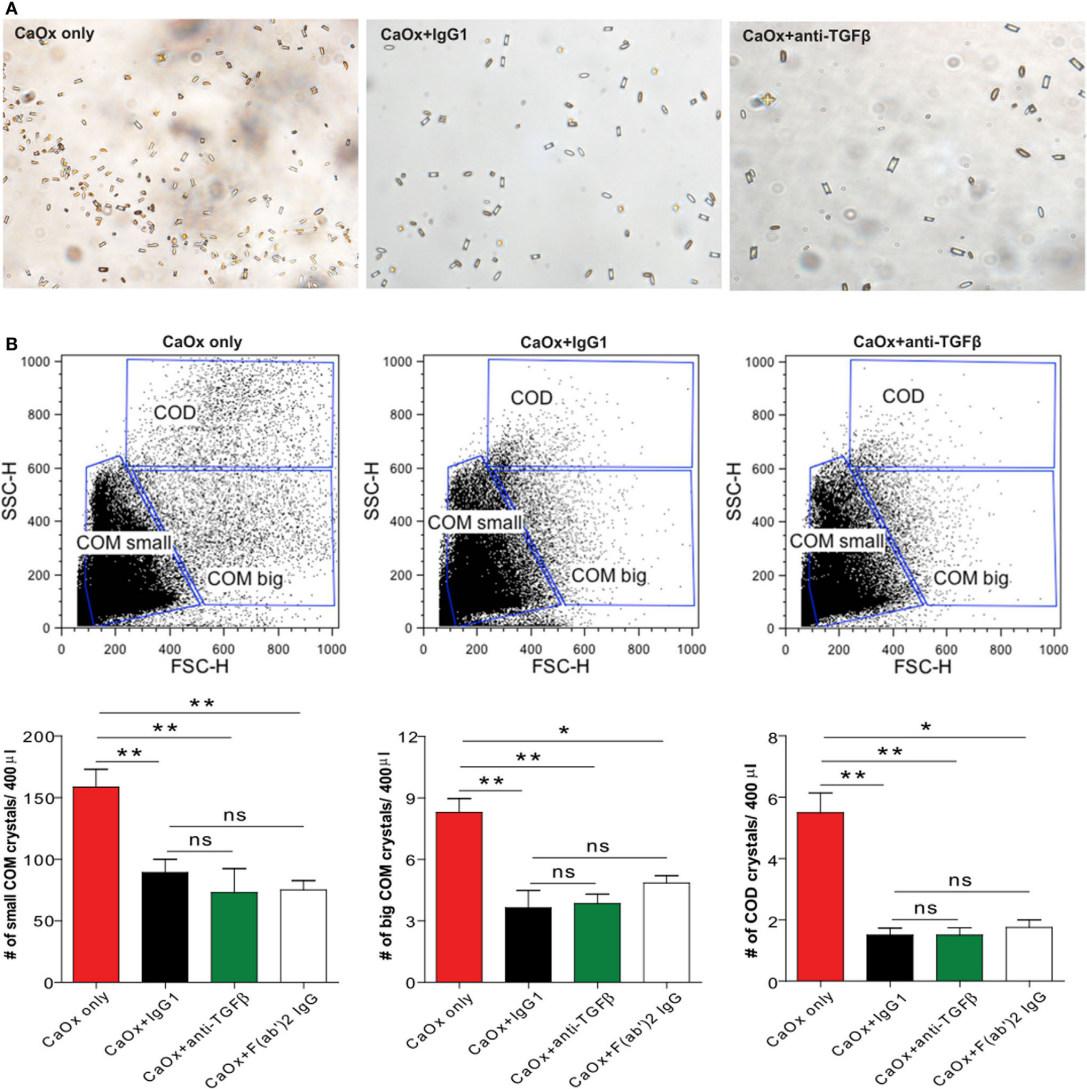
IgG1 affects calcium oxalate (CaOx) crystal formation *in vitro*. Na_2_C_2_O_4_ (oxalate) solution was pre-incubated with or without the control IgG1 (CaOx + IgG1) or anti-transforming growth factor (TGF)β IgG antibody (CaOx + anti-TGFβ) or an IgG F(ab′)2 fragment antibody [CaOx + F(ab′)2 IgG] for 1 h at room temperature and then CaCl_2_ solution added. After 5 min, CaOx monohydrate (COM) and CaOx dihydrate (COD) crystals were formed and visualized under the light microscope **(A)**. Quantification of COM and COD crystals by size (forward scatter vs. sideward scatter) using flow cytometry analysis **(B)**. Data are mean ± SEM (*n* = 5). *p < 0.05, **p < 0.01 are considered significant.

Calcium oxalate can crystallize in three different hydrate forms: COM, COD, and CaOx trihydrate crystals (7). Among these, COM crystals are the most common form of CaOx crystals present in urinary calculi in humans compared to COD crystals (23, 24). To investigate whether a monoclonal anti-TGFβ IgG antibody can directly influence CaOx crystal formation, we pre-incubated Na_2_C_2_O_4_ (sodium oxalate) with the anti-TGFβ IgG or the control IgG1 antibody followed by addition of CaCl_2_ (calcium chloride) to form CaOx crystals *in vitro*. Light microscopy revealed that COM crystals were predominantly formed whereas in the presence of anti-TGFβ or IgG1 the number of CaOx crystals decreased compared to CaOx only (Na_2_C_2_O_4_ + CaCl_2_ only) (Figure 1A). Using flow cytometry analysis, we identified small and big COM crystals as well as COD crystals depending on their size (side scatter vs. forward scatter), which in numbers significantly decreased upon pre-incubation with the anti-TGFβ or IgG1 antibody compared to CaOx crystals only (Figure 1B). Pre-incubated of sodium oxalate with only an IgG F(ab′)2 fragment prior to addition of calcium chloride revealed that the number of small and big COM crystals as well as COD crystals significantly decreased compared to CaOx alone (Figure 1B, white bars). However, no difference in the CaOx crystal formation was observed between pre-incubation with the anti-TGFβ or IgG1 antibody and the IgG F(ab′)2 fragment (Figure 1B). To rule out the possibility that IgG1 could interfere with soluble oxalate, we used microscale thermophoresis, a sensitive method that enables the quantitative analysis of molecular interactions in solution based on the movement of molecules along temperature gradients (25–27). Incubation of soluble oxalate with IgG1 revealed that soluble oxalate did not bind to IgG1 (data not shown). These findings indicate that IgG antibodies and even only the IgG F(ab′)2 fragment can directly affect CaOx crystal formation *in vitro*.

### IgG1 Antibody Treatment Ameliorates Renal Outcome in Chronic Oxalate Nephropathy *In Vivo*

**Figure 2:**
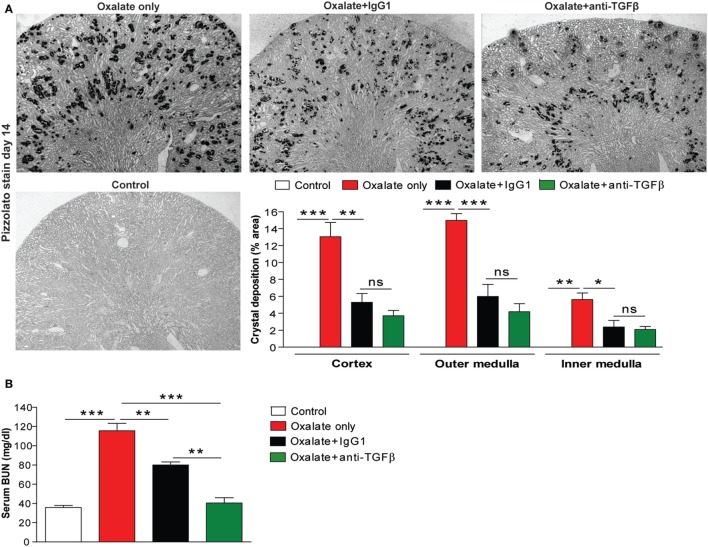

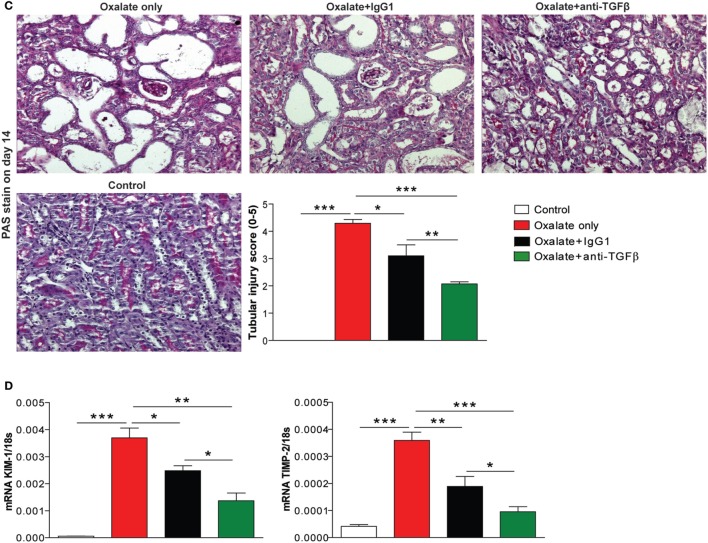
IgG antibody treatment improves renal outcome in CaOx crystal-induced injury *in vivo*. **(A)** Mice were either fed an oxalate-rich diet for 14 days (oxalate only) or were additionally injected with an IgG1 (oxalate + IgG1) or anti-transforming growth factor (TGF)β IgG antibody (oxalate + anti-TGFβ) or fed a control diet (control) (total 7 i.p. injections). **(A)** Quantification of CaOx crystal deposition (% area) in cortex, outer and inner medulla of Pizzolato stained kidney sections from the four groups on day 14. **(B)** Renal functional parameter serum blood urea nitrogen (BUN) levels on day 14. **(C)** Periodic acid-Schiff (PAS) staining illustrating tubular injury (original magnification 20×) and quantification of the tubular injury score. **(D)** Intrarenal mRNA expression of the kidney injury marker (KIM)-1 and tissue inhibitor of metalloproteinase (TIMP)-2. Data are mean ± SEM from five mice per group out of two independent experiments. ns, not significant. *p < 0.05, **p < 0.01, ***p < 0.001 are considered significant.

To investigate whether the effect of anti-TGFβ IgG antibody treatment on CaOx crystal formation also applies *in vivo*, we used a previously characterized mouse model of CaOx crystal-induced nephropathy (13). Feeding mice a high-oxalate diet resulted in the deposition of CaOx crystals in the cortex, as well as the outer and inner medulla compared to mice receiving the control diet, as illustrated by the % area of CaOx crystal deposition in Figure 2A (red bars). Early administration of hyperoxaluric mice with the anti-TGFβ (oxalate + anti-TGFβ) or control IgG1 antibody (oxalate + IgG1) significantly reduced intrarenal CaOx crystal deposits compared to untreated mice with nephrocalcinosis (oxalate only) after 14 days (Figure 2A). Serum as well as urinary oxalate levels significantly increased following oxalate feeding, whereas antibody treatment significantly reduced oxaluria in mice with nephrocalcinosis (Figure S1 in Supplementary Material). Urinary calcium levels rather declined upon feeding a calcium-depleted diet without being affected by antibody treatment (Figure S1 in Supplementary Material). Water intake was 0.2 ml/g BW in the control group (*n* = 5 per cage) and 0.29 ml/g BW in the oxalate only group (*n* = 5 per cage) on day 14 (data not shown).

Preemptive anti-TGFβ and IgG1 antibody treatment improved renal function, as indicated by decreased serum BUN levels (Figure 2B) compared to animals receiving a high-oxalate diet only after 14 days. This data was in line with progressive tubular atrophy and dilation in mice with nephrocalcinosis (oxalate only), which significantly decreased following preemptive administration of the antibodies, as indicated by PAS staining (Figure 2C) and intrarenal mRNA expression levels of the kidney injury marker-1 and tissue inhibitor of metalloproteinase-2 (Figure 2D). We did not observe any differences in the above measured parameters between the oxalate only and the antibody-treated groups on day 7 (data not shown). The data indicate that anti-TGFβ IgG prevents mice from CaOx crystal-induced nephropathy by influencing CaOx crystal formation.

### The Dual Effect of Anti-TGFβ IgG Attenuates CaOx Crystal-Related Tissue Inflammation

**Figure 3:**
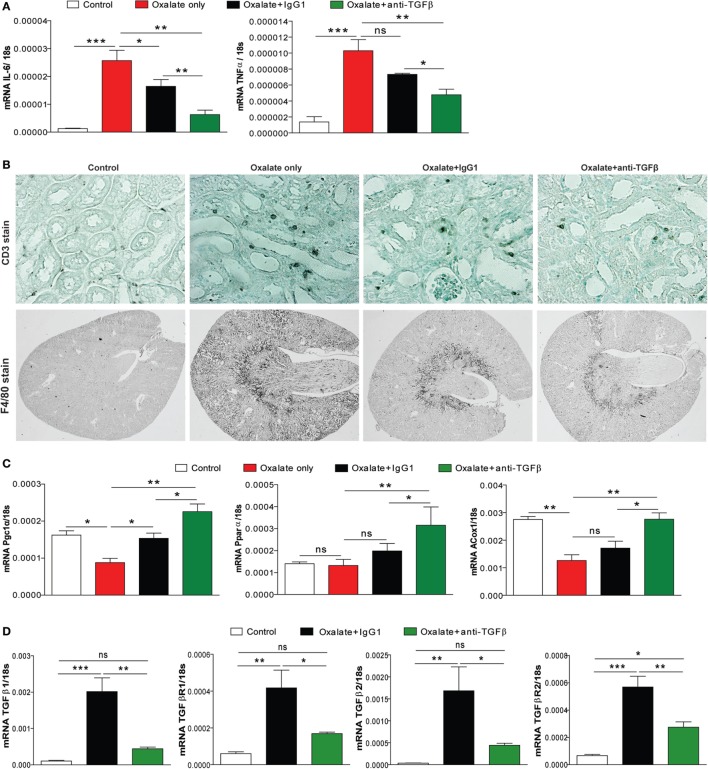
Anti-transforming growth factor (TGF)β IgG treatment ameliorates renal inflammation in chronic oxalate nephropathy. C57BL/6N mice were either fed an oxalate-rich diet for 14 days (oxalate only) or were additionally injected with an IgG1 (oxalate + IgG1) or anti-TGFβ antibody (oxalate + anti-TGFβ) or fed a control diet (control) (total 7 i.p. injections). **(A)** Intrarenal mRNA expression of the inflammatory cytokines interleukin (IL)-6 and tumor necrosis factor (TNF)α. **(B)** Immunostaining illustrating infiltrating CD3+ T cells and F4/80+ macrophages on kidney sections. **(C,D)** Intrarenal mRNA of the fatty acid enzymes peroxisome proliferator-activated receptor gamma coactivator 1 alpha (PGC1α), peroxisome proliferator-activated receptor alpha (PPARα), and peroxisomal acyl-coenzyme A oxidase 1 (ACOX1) **(C)**, and of the TGFβ signaling-related genes TGFβ1 and TGFβ2 as well as the receptors TGFβR1 and TGFβR2 **(D)** was performed on kidney RNA isolates. Data are mean ± SEM from five mice per group out of two independent experiments. ns, not significant. *p < 0.05, **p < 0.01, ***p < 0.001 are considered significant.

Given the potential of CaOx crystals to induce tubular atrophy, we next looked at the effect of anti-TGFβ IgG antibody therapy on the inflammatory response. As shown in Figure 3A, expression profiling of the inflammatory mediators interleukin 6 and tumor necrosis factor α showed a significant increase in the mRNA levels in mice with nephrocalcinosis (oxalate only) compared to the control group on day 14 (Figure 3A). This inflammatory response was significantly reduced in IgG1 antibody-treated mice and even further in mice treated with anti-TGFβ IgG (Figure 3A). Nephrocalcinosis-related progressive CKD is associated with increasing infiltration of immune cells, e.g., macrophages into the renal interstitial compartment (13, 28). Using immunostaining of kidney sections, we observed that anti-TGFβ and IgG1 antibody treatment reduced the number of intrarenal CD3+ T cells and F4/80+ macrophages compared to mice with nephrocalcinosis (Figure 3B).

In a recent study, alterations in the energy metabolism including fatty acid oxidation have been observed in kidneys from human subjects with CKD and in mouse models of kidney fibrosis (29). They also found that high levels of TGFβ during renal fibrosis play an important role in inhibiting fatty acid oxidation and thereby aggravating disease progression (29). We, therefore, examined the effect of anti-TGFβ treatment on the fatty acid metabolism. Intrarenal mRNA expression profiling revealed that peroxisome proliferator-activated receptor gamma coactivator 1 alpha, peroxisome proliferator-activated receptor alpha, and peroxisomal acyl-coenzyme A oxidase 1 were reduced in mice with nephrocalcinosis (oxalate only, red bars) (Figure 3C). Interestingly, anti-TGFβ treatment restored the renal energy metabolism compared to the oxalate only group (Figure 3C). We confirmed that neutralizing TGFβ *in vivo* inhibited TGFβ signaling as indicated by decreased intrarenal mRNA expression of TGFβ1 and TGFβ2 as well as the receptors TGFβR1 and TGFβR2 (Figure 3D). Together, the data indicate that anti-TGFβ IgG treatment prevents mice from CaOx crystal-induced inflammation but increased fatty acid oxidation.

### The Dual Effect of Anti-TGFβ IgG Reduces the Number of Pro-Inflammatory Macrophages

**Figure 4:**
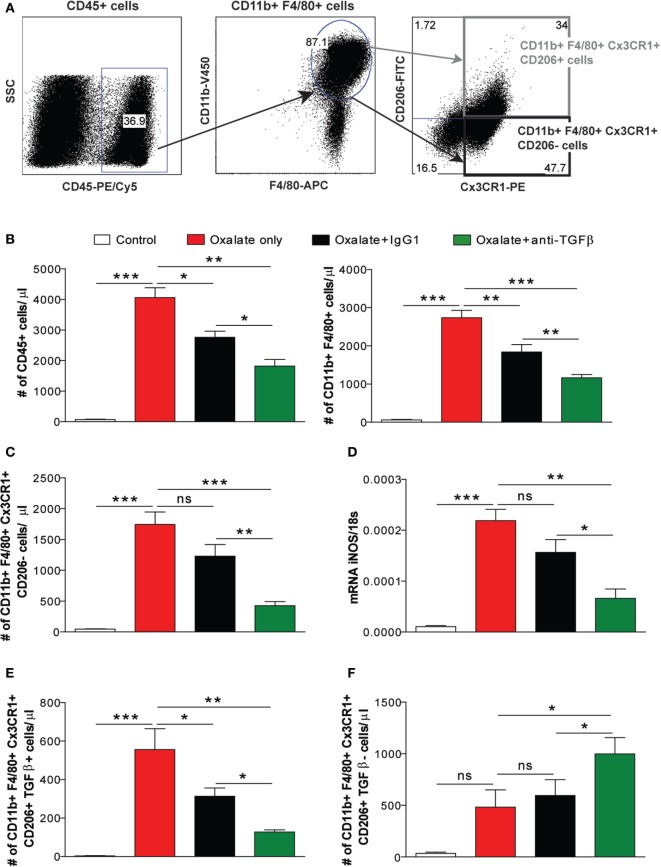
Treatment with the anti-transforming growth factor (TGF)β IgG antibody decreases nephrocalcinosis-related macrophage infiltrates. C57BL/6N mice were either fed a control diet, a high-oxalate diet only or combined with IgG1 or anti-TGFβ antibody treatment for 14 days. **(A,B)** Flow cytometric analysis of infiltrating CD45+ leukocytes and gating strategy of CD45+ F4/80+ CD11b+ macrophages in kidneys with absolute numbers. **(C)** Renal infiltrating macrophages identified as pro-inflammatory (M1-like) (CD45+ F4/80+ CD11b+ CX3CR1+ CD206−) macrophages with absolute cell numbers. **(D)** mRNA expression of inducible nitric oxide synthase (iNOS) was performed on kidney RNA isolates. **(E,F)** Absolute cell numbers of pro-fibrotic (M2a-like) macrophages (CD45+ F4/80+ CD11b+ CX3CR1+ CD206+ TGFβ+) **(E)** and anti-inflammatory (M2c-like) macrophages (CD45+ F4/80+ CD11b+ CX3CR1+ CD206+ TGFβ−) **(F)** in the kidneys. Data are mean ± SEM from six to seven mice per group out of two independent experiments. ns, not significant. *p < 0.05, **p < 0.01, ***p < 0.001 are considered significant.

Different macrophage phenotypes are associated with either the resolution of inflammation and tissue regeneration or persistent injury and progression to tissue atrophy, whereby their heterogeneity is determined by the microenvironment (30–32). We, therefore, carried out flow cytometry analysis to understand the diversity of phenotypes among the infiltrating macrophages. We noted that nephrocalcinosis is associated with increased numbers of CD45+ leukocytes in IgG1-treated mice compared to the control group (Figures 4A,B). However, anti-TGFβ IgG treatment significantly reduced the number of CD45+ leukocytes in mice with nephrocalcinosis (Figure 4B). The infiltrating macrophages were identified as CD45+ F4/80+ CD11b+ (Figure 4B). Phenotype analysis of macrophages revealed that these were pro-inflammatory (M1-like) macrophages (CD45+ F4/80+ CD11b+ CX3CR1+ CD206−) and M2-like macrophages (CD45+ F4/80+ CD11b+ CX3CR1+ CD206+) (Figure 4A) (30). Upon anti-TGFβ antibody treatment, the number of renal pro-inflammatory macrophages was significantly reduced compared to IgG1-treated or untreated mice with nephrocalcinosis (Figure 4C), which was consistent with less intrarenal mRNA expression of inducible nitric oxide synthase (iNOS), a mediator of inflammatory responses (Figure 4D). This indicates that anti-TGFβ treatment reduces the number of pro-inflammatory (M1-like) macrophages during nephrocalcinosis.

### The Dual Effect of Anti-TGFβ IgG Suppresses Nephrocalcinosis-Related Interstitial Fibrosis

**Figure 5:**
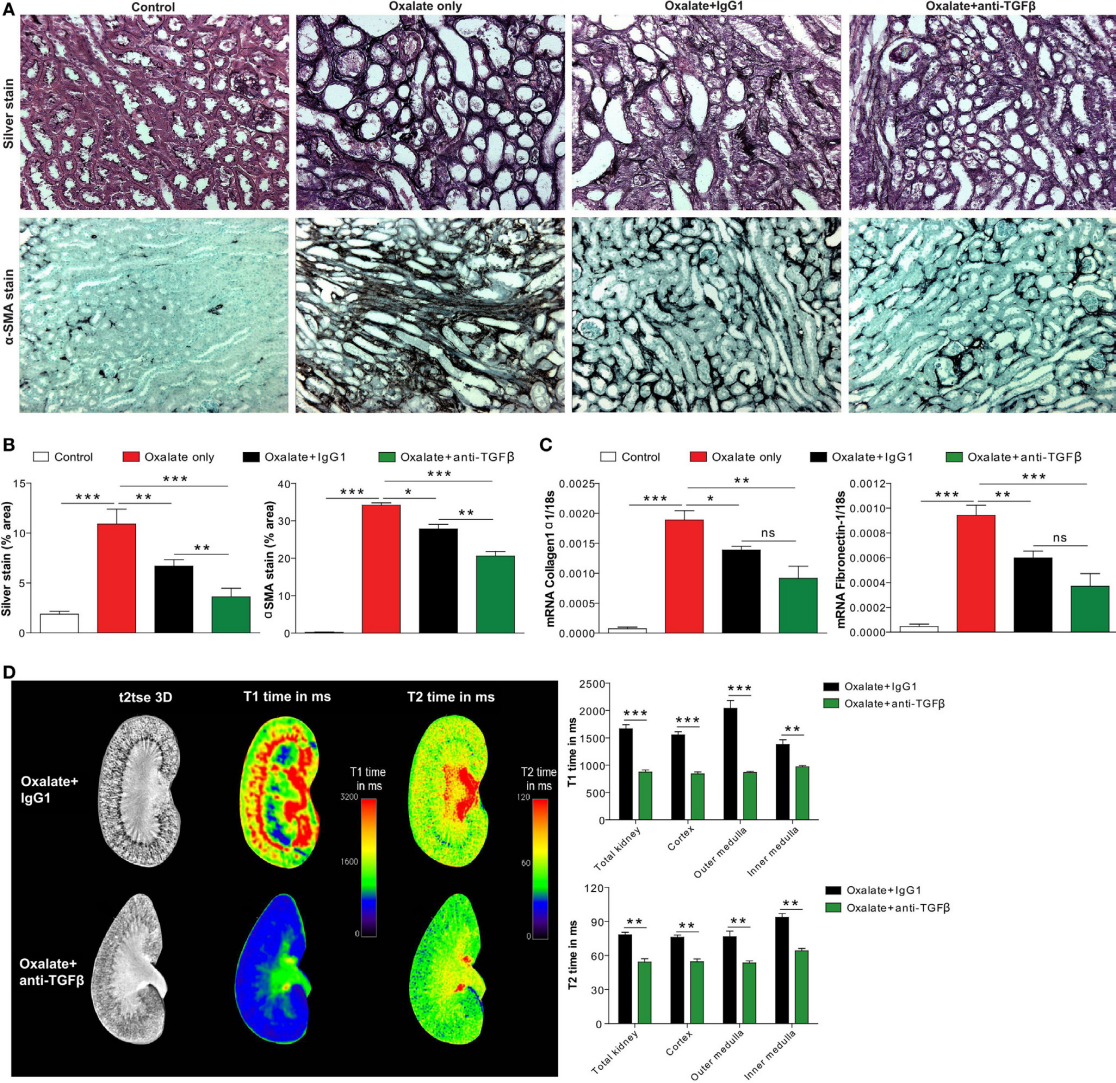
Neutralizing transforming growth factor (TGF)β inhibits nephrocalcinosis-related fibrosis. C57BL/6N mice were fed a high-oxalate diet only or in combination with preemptive IgG1 or anti-TGFβ antibody treatment for 14 days. **(A,B)** Immunohistochemistry images **(A)** and quantification of silver and alpha smooth muscle actin (αSMA) staining **(B)** was performed on kidney sections. **(C)** mRNA expression of collagen1α1 and fibronectin-1 was performed on kidney RNA isolates. **(D)** Magnetic resonance imaging of kidneys of IgG1 or anti-TGFβ antibody-treated mice with nephrocalcinosis. Representative images and quantification of T1 and T2 relaxation interval time showed significant differences in inflammation and fibrosis between the two groups. Data are mean ± SEM from five mice in each group. ns, not significant. *p < 0.05, **p < 0.01, ***p < 0.001 are considered significant.

Progressive nephrocalcinosis is associated with diffuse interstitial fibrosis, a process widely thought to contribute to CKD worsening (13, 33). We next investigated the impact of anti-TGFβ antibody treatment on interstitial fibrosis during chronic CaOx crystal-induced nephropathy. Immunohistochemistry staining of kidney sections for silver and αSMA revealed an increase of fibrotic lesions in mice with nephrocalcinosis as demonstrated by % area (Figures 5A,B). Following anti-TGFβ or IgG1 antibody treatment, however, we observed significantly less fibrosis (Figures 5A,B), which was in line with decreased intrarenal mRNA expression levels of the fibrosis marker collagen-1α1 and fibronectin-1 in the anti-TGFβ antibody treatment compared to the oxalate only group (Figure 5C). Further flow cytometry analysis showed that the number of pro-fibrotic (M2a-like) macrophages (CD45+ F4/80+ CD11b+ CX3CR1+ CD206+ TGFβ+) (34) significantly decreased (Figure 4E), whereas the number of anti-inflammatory (M2c-like) macrophages (CD45+ F4/80+ CD11b+ CX3CR1+ CD206+ TGFβ−) increased (Figure 4F) upon anti-TGFβ treatment in mice with nephrocalcinosis. In addition, we performed MRI of kidneys from mice with nephrocalcinosis. Compared to mice given the IgG1 antibody, animals receiving the anti-TGFβ antibody displayed a significant decrease in T1 and T2 time interval in the MRI analysis, suggesting less renal inflammation and fibrosis following TGFβ neutralization in mice with nephrocalcinosis (Figure 5D). Together, inhibiting fibrosis attenuates nephrocalcinosis-related CKD in association with a shift from pro-inflammatory (M1-like) and pro-fibrotic (M2a-like) macrophages toward an anti-inflammatory (M2c-like) macrophage phenotype.

### Preemptive but Not Delayed-Onset Inhibition of TGFβ-Mediated Interstitial Fibrosis Prevents Progressive GFR Decline

**Figure 6:**
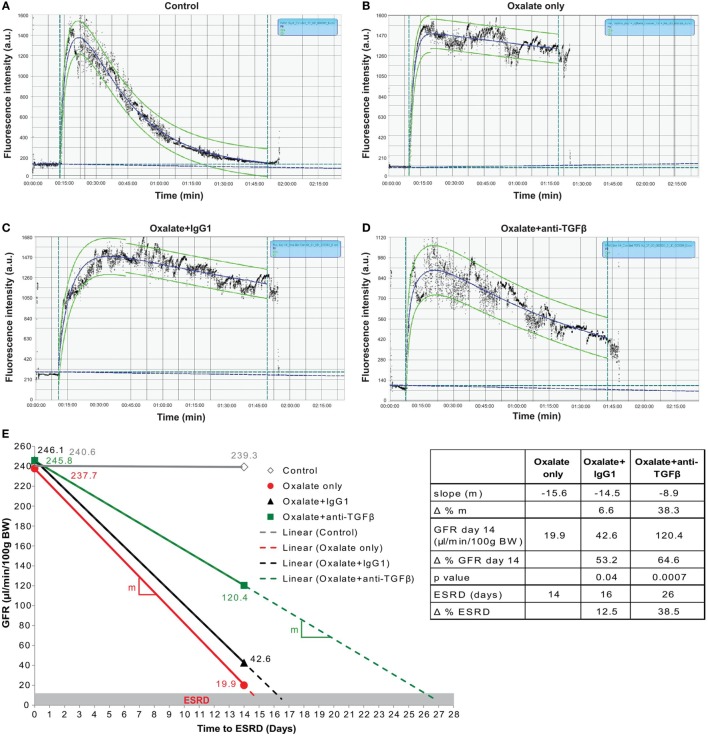
Preemptive transforming growth factor (TGF)β neutralization attenuates progressive glomerular filtration rate (GFR) decline. C57BL/6N mice were either fed a control diet (control), a high-oxalate diet (oxalate only) or an oxalate-rich diet combined with preemptive IgG1 or anti-TGFβ antibody treatment (oxalate + IgG1 and oxalate + anti-TGFβ) for up to 14 days. **(A–D)** Representative images of FITC-sinistrin fluorescence intensity curves using the three-compartment model with linear correction from a healthy mouse (control) **(A)**, a mouse with nephrocalcinosis **(B)**, an IgG1-treated mouse **(C)**, or anti-TGFβ antibody-treated mouse **(D)** with nephrocalcinosis. **(E)** GFR decline of all four groups from day 0 to 14 illustrated by the effect sizes: linear equation with slope (*m*, colored lines), GFR on day 14, and time to end stage renal disease (ESRD) (dotted lines and table). Δ represents difference expressed in percent (%). Data are mean ± SEM from five to seven mice per group and representative of one out of two independent experiments.

To study the impact of less interstitial fibrosis on progressive GFR decline, we repetitively measured GFR in mice on oxalate diet (day 0 and 14) or control diet (day 14). The GFR measurement process relies on a percutaneous acquisition of the clearance kinetics of a fluorescent signal emitted by the fluorescent tracer FITC-sinistrin upon bolus injection, a diagnostic tool in rodents (13, 35) and humans (36). We used a three-compartment kinetic model with linear correction to determine the GFR in mice. This model covers the injection process, the plasma volume, and the interstitial compartment allowing further refinement by correcting for shifts observed occasionally during measurements (37). After injecting FITC-sinistrin into a mouse that was fed a control diet (control) for 14 days, the fluorescent signal quickly increased at the beginning and descends down to the initial baseline level as it gets filtered by the kidneys after approximately 90 min. An example is illustrated in Figure 6A. The fluorescent signal of FITC-sinistrin was automatically recorded and the half-life (*t*1/2) determined (curve fitting 40% after peak signal, green dotted line) for calculating the GFR (21). Feeding mice an oxalate-rich diet resulted in an impaired renal clearance of FITC-sinistrin as indicated by the higher fluorescence intensity after 90 min (Figure 6B) compared to the FITC-sinistrin signal from a healthy mouse (Figure 6A). However, preemptive anti-TGFβ IgG therapy resulted in a decrease in the fluorescence intensity of FITC-sinistrin of mice with nephrocalcinosis after 90 min (Figure 6D) compared to an IgG1-treated mouse with nephrocalcinosis (Figure 6C).

Next, we calculated the three effect sizes: (1) the slope of daily GFR decline, (2) the GFR at the end of the study, and (3) the end stage renal disease (ESRD)-free renal survival. As illustrated in Figure 6E, the daily GFR loss was calculated using the linear equation (solid lines) between baseline (day 0) and day 14 indicating the slope (*m*) of the linear lines. Using this linear equation, we extended these lines (dotted lines) up to when they reached the GFR cut off of 15 μl/min/100 g BW, which we defined as ESRD (gray range). We found that the slope of daily GFR loss was *m* = −15.6 in mice with nephrocalcinosis (oxalate only, table and red line) compared to IgG1 treatment (*m* = −14.5) (Figure 6E, table and black line). However, preemptive TGFβ inhibition decreased the slope by Δ = 38.3% (*m* = −8.9) (Figure 6E, table and green line). At the end of the study on day 14, we observed a significant difference in the GFR in the IgG1-treated mice with an increase from 19.9 to 42.6 μl/min/100 g BW (Δ = 53.2%, *p* = 0.04) compared to oxalate-fed mice only (Figure 6E, table). This beneficial effect on the GFR on day 14 was further improved by treating mice with the anti-TGFβ IgG antibody (42.6–120.4 μl/min/100 g BW) (Δ = 64.6%, *p* = 0.0007) compared to IgG1-treated mice with nephrocalcinosis (Figure 6E, table). Assuming a linear GFR decline in the future, IgG1 treatment extended the ESRD-free renal survival compared to untreated mice with nephrocalcinosis by 2 days (Δ = 12.5%, black dotted line). Anti-TGFβ IgG therapy on the other hand prolonged the ESRD-free renal survival time even up to 10 days (Δ = 38.5%, green dotted line) compared to IgG1-treated mice.

In a clinical setting, anti-fibrotic treatments might be mainly considered upon detecting renal fibrosis in a diagnostic kidney biopsy but the window-of-opportunity for targeting renal fibrosis in CKD is unknown. Therefore, we explored the capacity of a delayed anti-TGFβ IgG treatment to improve GFR in mice with nephrocalcinosis by initiating anti-TGFβ or control IgG1 therapy from day 7, a time point when some tubular atrophy had already established (Figure S2 in Supplementary Material). Assessing the renal excretory function, we observed no protective effect of TGFβ inhibition with any of the aforementioned parameters (Figure S2 in Supplementary Material). Together, the data show that preemptive inhibition of TGFβ-driven interstitial fibrosis significantly preserves GFR decline and increased the ESRD-free renal survival of mice with progressive nephrocalcinosis-related CKD. However, delayed onset of TGFβ inhibition does no longer attenuate renal function decline or expand the ESRD-free renal survival.

## Discussion

We hypothesized that anti-TGFβ IgG antibody treatment may not only inhibit interstitial fibrosis but also influence the crystallization of CaOx inside the kidney, which both should synergize to prevent nephrocalcinosis-related GFR decline. We now show that the anti-TGFβ IgG antibody has a dual role by influencing the crystallization process of CaOx crystals and inhibiting interstitial fibrosis in a mouse model of progressive CaOx crystal-induced nephropathy.

In kidney stone disease or nephrocalcinosis, interactions between urinary constituents and CaOx crystals may influence one or more critical processes in the stone pathogenesis, including crystal nucleation, aggregation, growth, and adhesion of crystals and/or aggregates to the epithelial cell surface in the kidney (38). The mechanism of CaOx crystallization involves a combination of processes, including urine supersaturation of stone-forming salts, such as calcium and oxalate, urinary pH (6, 7, 39). A variety of urinary constituents have been identified as possible inhibitors of CaOx crystallization, growth, and cell membrane adhesion, in particular macromolecules (40–42), proteins (9, 10, 43–45), and phospholipids (46). Unlike healthy individuals, patients with CaOx nephrolithiasis or nephrocalcinosis show decreased urinary CaOx crystallization inhibitors. CaOx crystals can be coated with alternating electron-dense and light fibrils, or covered with a more amorphous granular material indicating binding of proteins to CaOx crystals (8, 47). Using a proteomics approach, Fong-ngern et al. identified a large number of apical proteins on distal renal tubular epithelial cells that can bind to COM crystals (48). To our knowledge, we report for the first time that an anti-TGFβ IgG and the control IgG1 antibody can influence CaOx crystal formation *in vitro* as well as *in vivo* using a mouse model of progressive CaOx crystal-induced nephropathy. We also found that the IgG F(ab′)2 fragment can influence CaOx crystallization *in vitro*. On the other hand, blocking TNFR signaling with Etanercept, a fusion protein consisting of an extracellular TNR receptor 2 and an Fc domain of the human IgG1, has been shown to reduce renal CaOx crystal deposition and prevent progressive nephrocalcinosis in mice (49). Thus, the exact mechanism of CaOx crystallization counteracting with IgG1 or the two main fragments is currently unknown and will need further investigation. So far, therapeutic approaches in humans primarily focus on reducing the risk of recurrent CaOx crystal formation (50) *via*, e.g., dietary restriction of oxalate-rich products (51), or reducing calcium and increasing citrate (52, 53).

Injury to every segment of the nephron can ultimately lead to loss of the entire nephron (54, 55). Toxic, inflammatory, or ischemic injury damages tubular cells leading to cell death, microvascular rarefaction, and fibroblast activation (56). Excessive myofibroblast accumulation of extracellular matrix in the interstitial and vascular compartment are accompanied by a significant decline in GFR and impaired epithelial regeneration (56). A variety of preclinical strategies to inhibit or even reverse interstitial fibrosis have been shown to be effective in rodents. For example, Kramann et al. demonstrated in two kidney fibrosis models that inhibiting the hedgehog pathway transcriptional effector GLI2 reduced renal fibrosis by limiting myofibroblast proliferation (57). Our finding confirms that administration of an anti-TGFβ IgG antibody can effectively prevent interstitial fibrosis in progressive CKD (16, 19, 58, 59). However, we did not observe a preservation of renal function upon delayed anti-TGFβ IgG antibody treatment. In addition, overexpression of latent TGFβ1 was shown to decrease both SMAD2/3 activation (transcription factors of canonical TGFβ1 signaling) and the number of myofibroblasts (60), which is in line with findings that SMAD3 knockout mice are protected from renal fibrosis (61, 62). Recent data also highlight a role for non-canonical TGFβ1 signaling *via* the transcription factor p53, a regulator of pro-fibrotic gene expression and cell cycle control in tubular epithelial cells (63–65), and for the complement factor C5 as therapeutic targets for fibrosis-related CKD (66). Strategies and efficacy of pharmacological therapies to reduce CKD progression are of upmost need (67). A dual-specific antibody approach for TGFβ neutralization successfully attenuated fibrosis in a mouse model of UUO (68). On the other hand, blocking TGFβ and Wnt-activated β-catenin crosstalk in proximal tubules enhances progressive CKD (69), suggesting that TβRII activity and the lack of proximal tubules stability may exacerbate fibrosis (70). Other than determining serum BUN and creatinine levels as well as other excretory markers of renal function, these animal studies did not assess GFR as primary endpoint. In our preclinical study, we used the transcutaneous GFR system based on the fluorescent tracer FITC-sinistrin, which is a precise method for repeated measurements in conscious and unrestricted mice to calculate the GFR (21, 22, 35, 37). Fresolimumab, a TGFβ neutralizing antibody did not elicit any positive effect on the GFR in a randomized control trial including 416 patients with diabetic kidney disease (71). Moreover, the anti-fibrotic agent pirfenidone was reported to consistently attenuate the GFR decline in mice and humans (72, 73).

In summary, anti-TGFβ IgG antibody treatment inhibits both CaOx crystallization and interstitial fibrosis in a model of CaOx crystal-induced nephropathy. Blocking TGFβ significantly improved the GFR decline by 38.3%, increased the GFR at the end of the study by 64.6%, and prolonged the time to ESRD by 38.5% compared to IgG1 control treatment. However, this nephroprotective effect got lost upon delayed onset of TGFβ blockade. We conclude that anti-TGFβ IgG antibody treatment elicits a dual effect on CaOx crystallization and interstitial fibrosis, which represents a novel therapeutic approach to delay progressive nephrocalcinosis-related CKD.

## Ethics Statement

All animal experiments were performed in accordance with the European protection law of animal welfare and were approval by the local government authorities Regierung von Oberbayern (reference number: 55.2-1-54-2532-189-2015).

## Author Contributions

SS and H-JA designed the study; SS, JG, QM, JJ, MS, and CB performed the experiments; SS, JG, QM, TB, JJ, MS, and ML analyzed and interpreted the data; SS and H-JA wrote the manuscript. All authors reviewed the manuscript.

## Conflict of Interest Statement

The authors declare that the research was conducted in the absence of any commercial or financial relationships that could be construed as a potential conflict of interest.

## Supplementary Material

Figure S1 Anti-TGFβ IgG treatment decreases oxaluria but has no effect on calcinuria. C57BL/6N mice were either fed a control diet (control), a high-oxalate diet (oxalate only) or an oxalate-rich diet combined with preemptive IgG1 or anti-TGFβ antibody treatment (oxalate + IgG1 and oxalate + anti-TGFβ) for up to 14 days. Serum and urine oxalate levels **(A)** and urine calcium levels **(B)** on day 14. Data are mean ± SEM from five mice per group. ns, not significant. **p* < 0.05, ***p* < 0.01, ****p* < 0.001 are considered significant.

Figure S2 Delayed anti-TGFβ IgG treatment does not improve renal function in chronic oxalate nephropathy. **(A)** Flow diagram of experimental design: C57BL/6N mice were fed an oxalate-rich diet combined with delayed IgG1 or anti-TGFβ treatment (total of 4 i.p. injections) (oxalate + IgG1 or oxalate + anti-TGFβ) compared to control diet (control) for 14 days. **(B–D)** Renal function was assessed by determining the serum blood urea nitrogen (BUN) levels **(B)**, tubular injury by periodic acid-Schiff (PAS) staining **(C)**, and measuring the glomerular filtration rate (GFR) **(D)** on day 14. Data are mean ± SEM from five mice per group. ns, not significant. **p* < 0.05, ***p* < 0.01, ****p* < 0.001 are considered significant.
