# Supplementary figures and images for: Anti-Transforming Growth Factor β IgG Elicits a Dual Effect on Calcium Oxalate Crystallization and Progressive Nephrocalcinosis-Related Chronic Kidney Disease
Source: Front Immunol. 2018 Mar 29;9:619. doi: 10.3389/fimmu.2018.00619 (PMC5884871; doi:10.3389/fimmu.2018.00619)

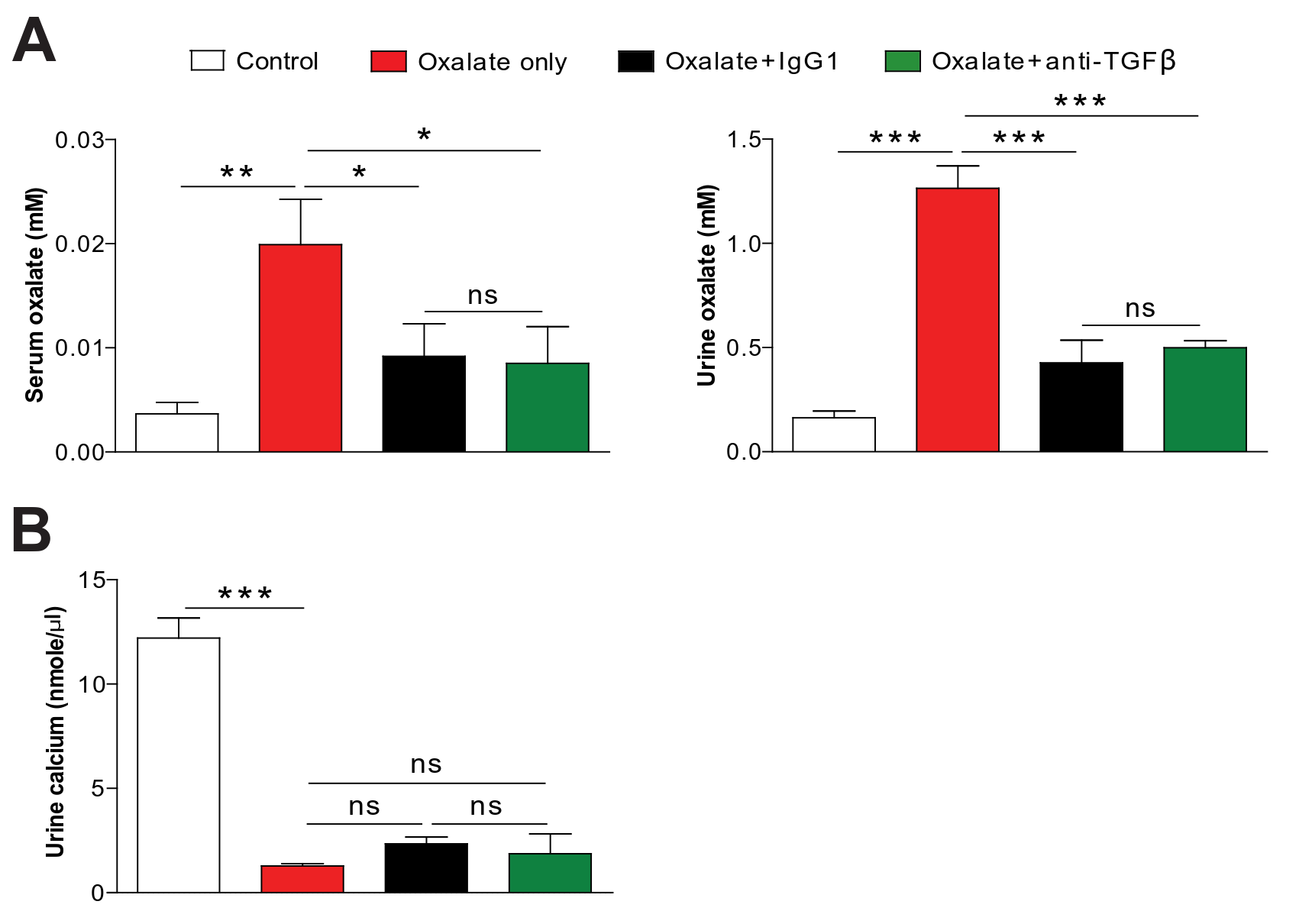

Supplement: Figure S1 — Anti-TGFβ IgG treatment decreases oxaluria but has no effect on calcinuria. C57BL/6N mice were either fed a control diet (control), a high-oxalate diet (oxalate only) or an oxalate-rich diet combined with preemptive IgG1 or anti-TGFβ antibody treatment (oxalate + IgG1 and oxalate + anti-TGFβ) for up to 14 days. Serum and urine oxalate levels (A) and urine calcium levels (B) on day 14. Data are mean ± SEM from five mice per group. ns, not significant. *p < 0.05, **p < 0.01, ***p < 0.001 are considered significant. [file image_1.PNG]

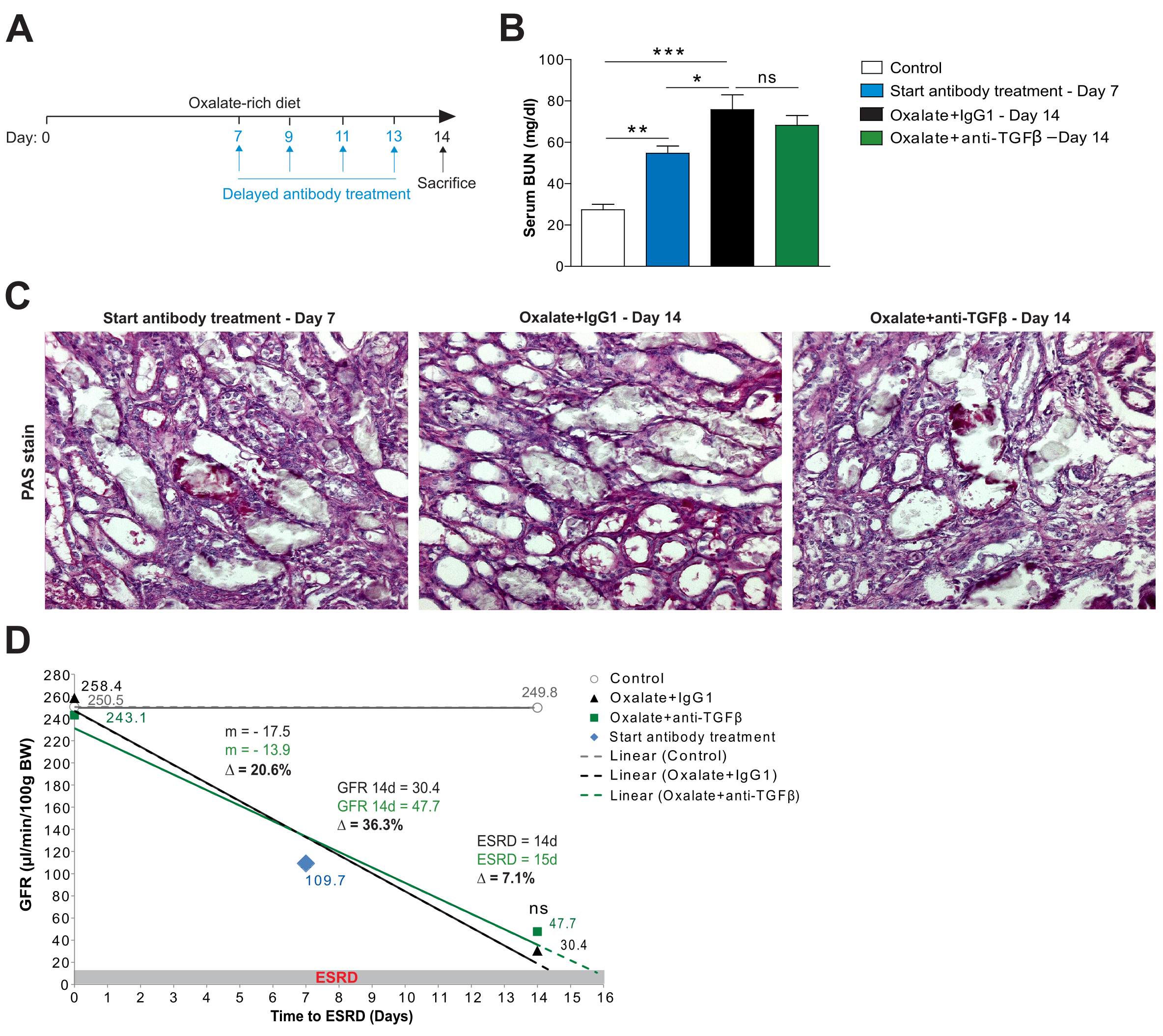

Supplement: Figure S2 — Delayed anti-TGFβ IgG treatment does not improve renal function in chronic oxalate nephropathy. (A) Flow diagram of experimental design: C57BL/6N mice were fed an oxalate-rich diet combined with delayed IgG1 or anti-TGFβ treatment (total of 4 i.p. injections) (oxalate + IgG1 or oxalate + anti-TGFβ) compared to control diet (control) for 14 days. (B–D) Renal function was assessed by determining the serum blood urea nitrogen (BUN) levels (B), tubular injury by periodic acid-Schiff (PAS) staining (C), and measuring the glomerular filtration rate (GFR) (D) on day 14. Data are mean ± SEM from five mice per group. ns, not significant. *p < 0.05, **p < 0.01, ***p < 0.001 are considered significant. [file image_2.PNG]
